# Ultrasound Depolymerization and Characterization of Poly- and Oligosaccharides from the Red Alga *Solieria chordalis* (C. Agardh) J. Agardh 1842

**DOI:** 10.3390/md22080367

**Published:** 2024-08-13

**Authors:** Mathilde Lesgourgues, Thomas Latire, Nolwenn Terme, Philippe Douzenel, Raphaël Leschiera, Nicolas Lebonvallet, Nathalie Bourgougnon, Gilles Bedoux

**Affiliations:** 1 Laboratoire de Biotechnologie et Chimie Marines (LBCM), EMR CNRS 6076, IUEM, Université Bretagne Sud, 56000 Vannes, France; tlatire@uco.fr (T.L.); nterme@uco.fr (N.T.); philippe.douzenel@univ-ubs.fr (P.D.); nathalie.bourgougnon@univ-ubs.fr (N.B.); gilles.bedoux@univ-ubs.fr (G.B.); 2 Laboratoire d’efficacité cosmétique (E-COS), Université Catholique de l’Ouest Bretagne Nord, 22200 Guingamp, France; 3 Laboratoire Interaction Epithéliums Neurones (LIEN), UR 4685, Université Bretagne Occidentale, 29200 Brest, France; raphael.leschiera@univ-brest.fr (R.L.); nicolas.lebonvallet@univ-brest.fr (N.L.)

**Keywords:** low-molecular-weight polysaccharide, radical depolymerization, iota-carrageenan, extraction, biorefinery, human dermal fibroblast

## Abstract

Red seaweed carrageenans are frequently used in industry for its texturizing properties and have demonstrated antiviral activities that can be used in human medicine. However, their high viscosity, high molecular weight, and low skin penetration limit their use. Low-weight carrageenans have a reduced viscosity and molecular weight, enhancing their biological properties. In this study, ι-carrageenan from *Solieria chordalis*, extracted using hot water and dialyzed, was depolymerized using hydrogen peroxide and ultrasound. Ultrasonic depolymerization yielded fractions of average molecular weight (50 kDa) that were rich in sulfate groups (16% and 33%) compared to those from the hydrogen peroxide treatment (7 kDa, 6% and 9%). The potential bioactivity of the polysaccharides and low-molecular-weight (LMW) fractions were assessed using WST-1 and LDH assays for human fibroblast viability, proliferation, and cytotoxicity. The depolymerized fractions did not affect cell proliferation and were not cytotoxic. This research highlights the diversity in the biochemical composition and lack of cytotoxicity of *Solieria chordalis* polysaccharides and LMW fractions produced by a green (ultrasound) depolymerization method.

## 1. Introduction

For many years, strandings of *Solieria chordalis* (C. Agardh) J. Agardh 1842 (Gigar-tinales, *Solieriaceae*) have been observed on the Rhuys Peninsula in southern Brittany (France). During autumn equinoctial storms, the algae are torn from their substrate and then, with the local winds and tides, they drift to the beaches. At over 2000 tons per year, these massive arrivals of seaweeds cause ecological and economic problems for the communities along the Brittany coast. To date, *S. chordalis* biomass is mainly used as fertilizer in the agricultural fields of the affected Brittany communes and in Algobox^®^, an environmental tool to protect the coast from erosion [1]. Several studies on *S. chordalis* from Brittany have shown its potential for biotechnological development [2,3]. In plant health and agriculture studies, water extracts obtained after enzyme-assisted extraction (EAE) showed biostimulant and biosorbent properties [2]. Choulot et al. [3] also demonstrated the efficient process benefits of using EAE—in comparison with conventional water extraction—for producing enriched extracts containing macro- and microelements and plant growth regulators for plant growth and development. Different alcoholic extracts produced from *S. chordalis* showed cytotoxic and pro-apoptotic effects on several human leukemia T cell lines, Burkitt lymphoma, and non-small cell lung cancer [4]. The purification and characterization of these extracts suggested the presence of polyunsaturated fatty acids, terpenes, and pigments. Kendel et al. (2015) also showed that monogalactosyldiacylglycerols obtained from *S. chordalis* through a gravimetric method have in vitro antiproliferative activity against a human bronchopulmonary carcinoma cell line [5].

Like many Gigartinales, *S. chordalis* is rich in linear sulfated matricial cell wall polysaccharides (carrageenans). Carrageenans have alternating 4-linked α-D-galactopyranose or 3,6-anhydro α-D-galactopyranose and 3-linked β-D-galactopyranose units. They are classified according to the presence of the 3,6-anhydro-bridge on the 4-linked galactose residue and the position and number of sulfate groups [6]. *S. chordalis* is characterized by the dominant presence of ι-carrageenan 4-sulfate-galactose and 3,6-anhydrogalactose 2-sulfate (G4S-DA2S). Additionally, α- and γ-carrageenan have also been detected at very low levels [1,7]. Structural cell wall polysaccharides account for around 5–30% of the algal biomass on a dry weight basis. They are involved in mechanical strength and resistance, cell and tissue adhesion, morphogenesis, development, hydration, and the protection of seaweeds. Carrageenans have been shown to exhibit antiviral activity against a wide spectrum of viruses. Recently, Pliego-Cortés et al. [8,9] have added evidence of the potential in vitro antiviral activity of the carrageenan extracted from stranding biomasses of *S. chordalis* against herpes simplex virus type 1 and 2 and SARS-CoV-2. The carrageenan obtained by EAE or by using hot water was isolated by ethanol precipitation and purified using dialysis and chromatography. These various studies confirmed the potential of this species and its high added-value compounds, as well as its availability in large quantities. Nevertheless, it is still underexploited for use in human medicine. More than that, despite their powerful biological activities, the high molecular weight, low solubility, high viscosity, and poor tissue-penetrating ability of these polysaccharides have limited their potential application in humans. By reducing the complexity and limitations and increasing the solubility, low-molecular-weight (LMW) carrageenans could be used in various application areas [10,11].

In this study, we explored the potential of polysaccharides and LMWs produced from *Solieria chordalis* collected during two different seasons on the Rhuys Peninsula in southern Brittany. The polysaccharides produced using conventional hot water extraction were depolymerized using two methods: hydrogen peroxide and ultrasound treatments. A biochemical composition analysis and molecular weight distribution studies were performed using High-Pressure Size-Exclusion Chromatography (HPSEC), High-Performance Anion-Exchange Chromatography (HPAEC-PAD), and Fourier Transform Infrared (FTIR) spectroscopy. Finally, their effects on the viability and proliferation of human dermal fibroblast cells were evaluated.

## 2. Results

### 2.1. Chemical Characterization of Algal Material

*S. chordalis* was collected at two different times in order to determine its seasonal variation in terms of biochemical composition. The dry weight of *S. chordalis* was 12.24% and 12.84% for the samples collected in June 2022 and October 2021, respectively. The strong winds and high tides during this time caused large quantities of seaweed to be removed from their substrate and washed up on the beaches. June 2022 was chosen due to favorable weather conditions. The ash, neutral sugar, uronic acid, protein, and sulfate group contents expressed in percentage of dry weight (% dw) were determined for the *S. chordalis* collected in October 2021 (Sc1021) and June 2022 (Sc0622) (Table 1). The ash content in the June 2022 sample (45.00%) was higher than that of the October 2021 sample (38.15%). Conversely the algae collected in October contained more neutral sugars (34.36%) and proteins (14.95%), while sulfate groups and uronic acids were not significantly different. The algal material was richer in glucose (75.04% in the October 2021 sample and 87.93% in the June 2022 sample) than galactose (23.40% and 10.77%, respectively).

The monosaccharide compositions were determined using High-Performance Anion-Exchange Chromatography (HPAEC) coupled with pulsed amperometric detection (PAD). Only compounds detected and identified using known standards were measured and quantified as a percentage of the total identified monosaccharides. This analysis provided a better understanding of the nature of the polysaccharides present in the seaweed. The algal material was richer in glucose (75.04% in October 2021 sample and 87.93% in June 2022 sample) than galactose (23.40% and 10.77%, respectively). The October 2021 sample contained more glucuronic acid (1.06%) than the June 2022 sample (0.51%) whereas the opposite was noticed for the xylose content (0.03% for October 2021 sample vs. 0.34% for June 2022 sample).

### 2.2. Extraction and Characterization of Solieria chordalis Extracts

**Figure 1:**
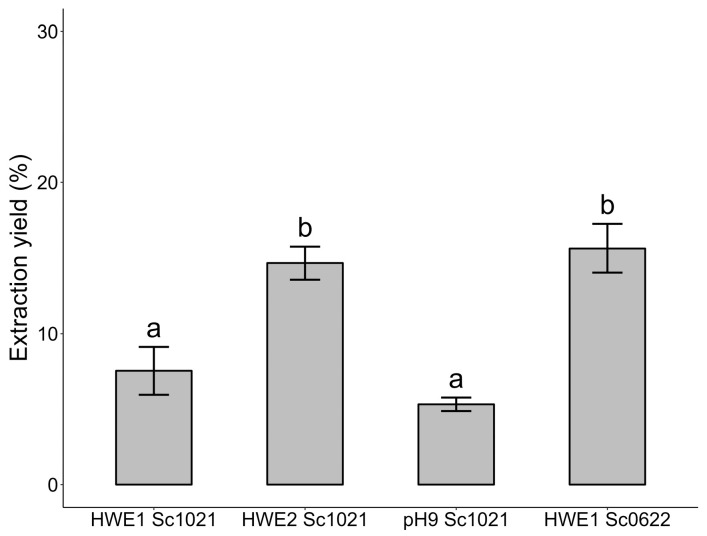
Yields (%) using different methods for extracting polysaccharides from the red alga *Solieria chordalis*. Different letters represent significant differences according to Tukey’s pairwise a posteriori test after ANOVA (*p* < 0.001). Sample size: *n* = 3.

The dry seaweed was depigmented and successively defatted in a Soxhlet extractor using acetone, ethanol/chloroform, and ethanol. Crude extracts were obtained after hot water extraction from the seaweeds collected in October 2021 (HWE1 Sc1021) and June 2022 (HWE1 Sc0622). A second extraction (HWE2 Sc1021) was performed on the residue obtained after the initial extraction (HWE1 Sc1021). From the sample collected in October, hot water extraction was also performed at pH 9. The extraction yields are summarized in Figure 1. The extraction yield was significantly higher for the seaweed collected in June (15.60%). The second extraction on the residue of HWE1 Sc1021 was able to isolate additional water-soluble compounds. The hot water extraction at pH 9 (5.32%) showed no significant difference compared to the hot water extraction performed at neutral pH (7.54%).

**Figure 2:**
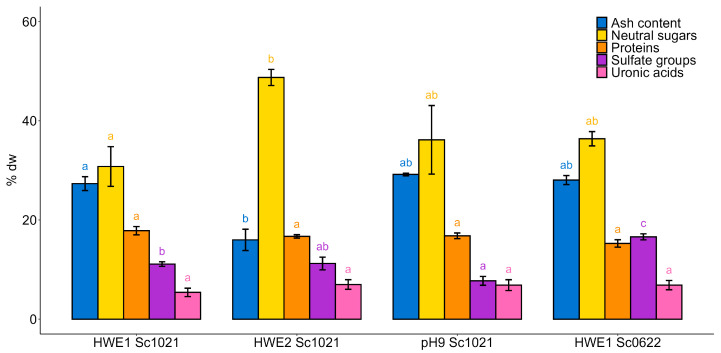
Chemical composition (%dry weight) of extracts obtained by different conventional hot water extraction methods from the red alga *Solieria chordalis*. Statistically significant differences are indicated by different letters from a to c (*p* < 0.05). Different letters represent significant differences according to Tukey’s pairwise a posteriori test after ANOVA. Sample size: *n* = 3.

The biochemical composition of the different extracts was characterized (Figure 2). Hot water extracts from the *S. chordalis* collected in June showed the highest amount of sulfate groups (16.60%), while hot water extracts from the *S. chordalis* collected in October did not exceed 12%. The protein content was not significantly different among the samples and ranged from 15.27% (HWE1 Sc0622) to 17.84% (HWE1 Sc1021). HWE2 Sc1021 showed the highest content of neutral sugars (48.73%), while its ash content was inferior at 16.00%. The extraction at pH 9 led to the lowest sulfate group content (7.73%). Analysis of the molecular weight profiles obtained by High-Pressure Size-Exclusion Chromatography (HPSEC) yielded a weight greater than 1.3 MDa for all extracts.

The monosaccharide composition of the extracts is presented in Table 2. Glucose and galactose were the main monosaccharides in all the samples. The xylose and glucuronic acid contents ranged from 0.42% to 5.44% and 2.95% to 9.67%, respectively. After hot water extraction, the ratio between glucose and galactose ranged between 0.79 (HWE1 Sc0622) and 4.04 (pH 9 Sc1021). HWE1 Sc1021 contained 60.24% glucose and 22.70% galactose (ratio of 2.65), while HWE2 Sc1021 contained 45.77% and 41.57%, respectively (ratio of 1.10). pH 9 Sc1021 showed a higher glucose content (74.04%) than galactose content (18.24%). In contrast, the fraction extracted from the seaweed collected in June (HWE1 Sc0622) contained more galactose (47.13%) than glucose (37.16%)

**Figure 3:**
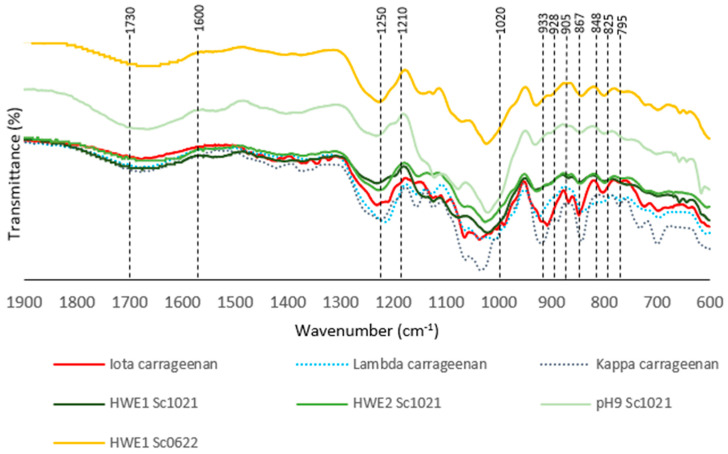
FTIR spectra of standards and extracts from *Solieria chordalis* after different hot water extraction procedures.

Based on the FTIR spectra of the hot water extracts, the samples were found to contain iota (ι)-type carrageenan by comparing to the commercial κ -, ι-, and λ-carrageenan spectra and the information available in the published literature on the same species. The FTIR spectra in the range of 1900 to 600 cm^−1^ were studied (Figure 3). Table 3 summarizes the characteristic vibrational and elongation bond wavenumbers observed from red algal galactanes, as well as the associated osidic subunits and polysaccharides [12,13,14,15]. All spectra (standards and samples) showed a large and intense band from sulfate esters at 1210–1250 cm^−1^. Signals corresponding to the linked C-O of the 3,6-anhydrogalactose ring were also detected at 1020 cm^−1^ and 928–933 cm^−1^ except in the λ-carrageenan standard spectrum. Additionally, the bands at 905 cm^−1^ and 795 cm^−1^, specific to the C-O-SO_3_ bond on C2 of 3,6-anhydrogalactose in ι-carrageenan, were present in the spectra for the corresponding standard and the samples. The 1600–1730 cm^−1^ interval contained broad, low-intensity bands due to the presence of C=O bonds, which are involved present in various functional groups such as aldehydes, ketones, esters, and carboxylic acids. The presence of these different specific signals enabled the identification of the presence of ι-carrageenan in the different fractions after extraction.

Significant stranding of *S. chordalis* occurs on Brittany’s coasts during October, providing a substantial biomass. That is why, based on the biochemical results discussed above (for neutral sugar and sulfate group contents and ι-type carrageenan) and the large available quantity of the October extract, HWE1 Sc1021 was used for purification and depolymerization assays in order to produce low-molecular-weight (LMW) carrageenans.

HWE1 Sc1021 was dialyzed with a 100 kDa cut-off threshold. Two phases were obtained, a supernatant (LDL-S 100) and a precipitate (LDL-P 100). The appearance of the precipitate may be due to the formation of protein–polysaccharide complexes linked to the elimination of salts, which promotes new electrostatic interactions during dialysis. An analysis of the biochemical composition of the two fractions showed that the dialysis process decreased the ash content to 14.34% in LDL-S 100 and 16.51% in LDL-P 100. An increase in the neutral sugar content was observed in LDL-S 100 (40.98%) in comparison with the crude extract HWE1 Sc1021, which contained 30.78% neutral sugars. Sulfate groups were present in similar quantities in the dialysis supernatant (9.68%) and in the crude extract (11.10%), while the dialysis precipitate contained fewer sulfate groups (7.40%). LDL-P 100 was higher in proteins (20.06%) than the initial extract (17.83%). The dialysis process did not affect the uronic acid content, which remained stable in the supernatant (5.95%) and precipitate (6.24%). However, an analysis of the monosaccharide compositions (Table S1) revealed a significant presence of glucose (46.48% in the supernatant and 61.91% in the precipitate) and galactose (32.71% and 21.46, respectively).

After dialysis of HWE1 Sc1021, the LDL-S 100 fraction contained polysaccharides with molecular weights above 1.3 MDa and the proportion of low-molecular-weight polysaccharides (Mw < 1.6 kDa) decreased after the dialysis process.

### 2.3. Study of Parameters Influencing Ultrasonic Depolymerization

HWE1 Sc1021 was depolymerized using ultrasound. To understand the impact of ultrasound depolymerization, a study of the influence of the time and amplitude was conducted. The final fractions were then biochemically characterized and their molecular weight profiles were also determined. In addition, the monosaccharide compositions were determined and are presented in Table S1.

#### 2.3.1. Effect of Amplitude

**Figure 4:**
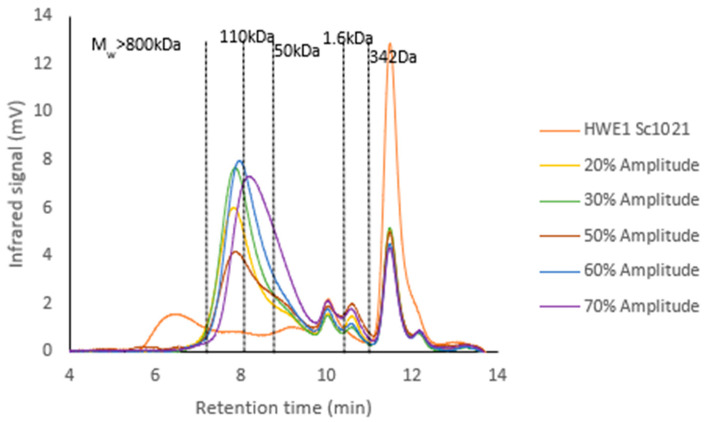
Chromatograms of the molecular weight profiles of HWE1 Sc1021 extract before and after depolymerization using different amplitudes.

HWE1 Sc1021 was depolymerized using ultrasound at 20, 30, 50, 60, and 70% amplitudes for 3 h. The application of increasing amplitude from 20% to 70% produced a decrease in the molecular weight (Figure 4). Amplitudes of 20% and 60% led to a molecular weight Mw > 300 kDa with a polydispersity index (PD) close to 1.6, indicating a homogeneous composition of the fraction. The results showed that the highest amplitude (70%) reduced the molecular weight of the initial fraction to 94 kDa but increased the polydispersity index to 3.53.

**Figure 5:**
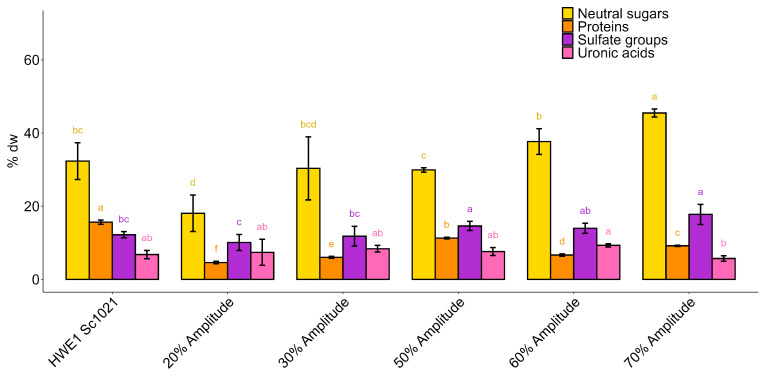
Biochemical composition (% dry weight) of fractions after depolymerization using ultrasound at different amplitudes. Statistically significant differences are indicated by different letters from a to d (*p* < 0.05). Different letters represent significant differences according to the Kruskal–Wallis a posteriori test. Sample size: *n* = 3.

The biochemical composition of the depolymerized fraction is summarized in Figure 5. The extract depolymerized at 20% amplitude showed a lower neutral sugar content (18.05%) in comparison with the initial fraction (32.31%). The fractions obtained after ultrasound treatment presented a neutral sugar content ranging from 18.05% to 45.46%. The protein levels were below 12% after the ultrasound treatment, which was lower than the protein level in the HWE1 Sc1021 extract (15.64%). The ultrasound depolymerization preserved the sulfate group content, which ranged from 10.08% to 17.77%, which was close or higher than that of the initial fraction (12.20%).

Increasing the amplitude resulted in a better reduction in molecular weight. However, amplitudes of 60% and 70% led to significant overheating of the probe and therefore, premature wear of the horn. In addition, the quantities of proteins, sulfate groups, and uronic acids obtained using amplitudes of 50%, 60%, and 70% were similar. Therefore, the use of ultrasonic treatment at 50% appeared to be the best amplitude for the following depolymerization experiments.

#### 2.3.2. Effect of Time

**Figure 6:**
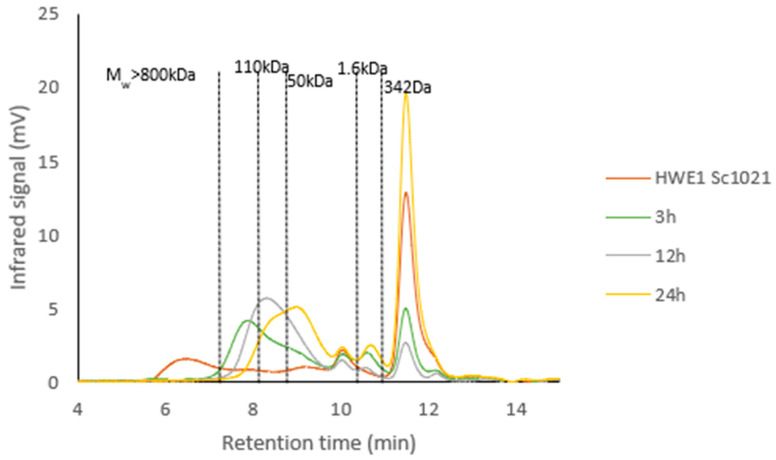
Chromatograms of the molecular weight profiles of HWE1 Sc1021 extract before and after depolymerization for different durations at 50% amplitude.

HWE1 Sc1021 was depolymerized using an ultrasound process for 3, 12, and 24 h at 50% amplitude. All three conditions reduced the molecular weight, resulting in fractions of 304 kDa, 85 kDa, and 23 kDa after 3, 12, and 24 h, respectively (Figure 6). Nevertheless, the polydispersity index (PD) was greater than 3 for the 12 h and 24 h samples in comparison with the 3 h depolymerization sample (PD = 1.60).

**Figure 7:**
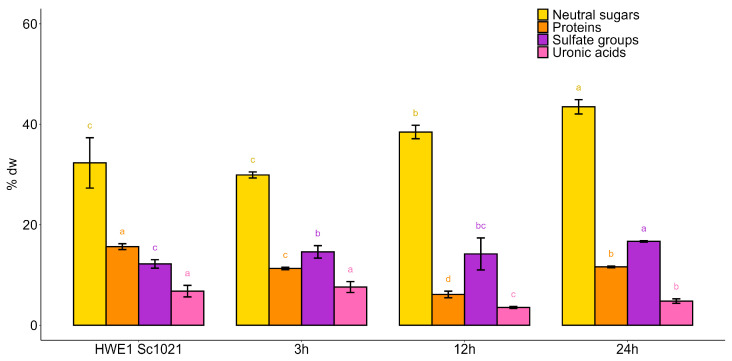
Biochemical composition of fractions after depolymerization using ultrasound for different durations. Statistically significant differences are indicated by different letters from a to d (*p* < 0.05). Different letters represent significant differences according to the Kruskal–Wallis a posteriori test. Sample size: *n* = 3.

The biochemical composition analysis showed that the ultrasonic depolymerization affected the neutral sugar content (Figure 7). Indeed, the ultrasonically depolymerized fractions exhibited a neutral sugar content exceeding 29%. The protein content was lower (ranging from 6.13% to 11.62%) compared to the polysaccharide fraction (15.64%) after the ultrasound treatment. Conversely, sulfate groups were well preserved after the ultrasound treatment, ranging from 14.19% to 16.69%. Furthermore, the sulfate content did not significantly differ between the 3 and 12 h of depolymerization samples (14.60% and 14.19%, respectively). However, after 24 h, the sulfate content was 16.69%.

Ultrasonic depolymerization for 24 h resulted in an average molecular weight of 23 kDa, and according to the biochemical composition, this corresponded to the best conditions. So, this condition was used for the following depolymerization experiments.

### 2.4. Depolymerization of HWE1 Sc1021 and LDL-S 100 Using Hydrogen Peroxide and Ultrasound

In the present study, the influence of hydrogen peroxide depolymerization parameters was not investigated, as this has already been studied using ulvans from a *Ulva* sp. in a previous study [16].

#### 2.4.1. HWE1 Sc1021 Depolymerization

**Figure 8:**
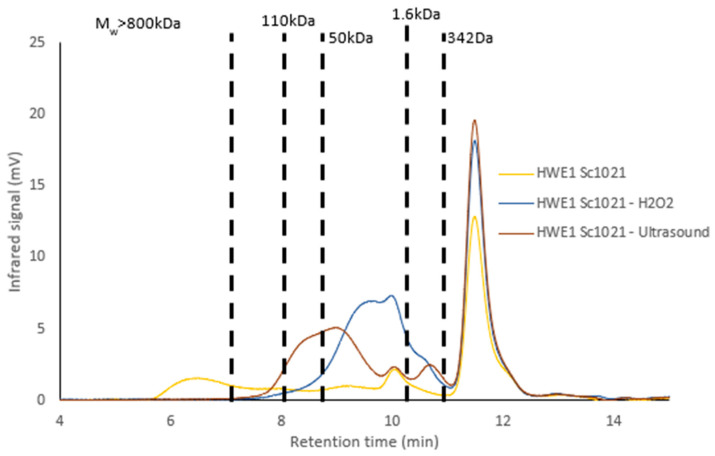
Molecular weight profile of HWE1 Sc1021 before and after depolymerization using H_2_O_2_ and ultrasound.

HWE1 Sc1021 was depolymerized by radical depolymerization using H_2_O_2_ (50 °C for 24 h) and ultrasound methods (50% amplitude for 24 h). The biochemical composition of the extracts was analyzed (Table 4) as well as the monosaccharide compositions and weight profiles (Figure 8).

Ultrasound depolymerization yielded a fraction with a higher sulfate content compared to the fraction obtained through radical depolymerization. Glucose and galactose were still the major monosaccharides present in the depolymerized samples (ranging from 66.59% to 67.19% and from 18.71% to 18.81%, respectively). The infrared spectra after depolymerization still showed the characteristic signals of ι-carrageenan, confirming its presence.

HWE1 Sc1021 contained polysaccharides with molecular weights above 1.3 MDa, as well as low-molecular-weight polysaccharides. Radical depolymerization using H_2_O_2_ produced a low-molecular-weight fraction (Mw = 7.6 kDa) with a low polydispersity (PD = 1.4), indicating a homogeneous size profile. Moreover, ultrasound depolymerization led to a final molecular weight of 23.6 kDa (PD = 3.30).

#### 2.4.2. LDL-S 100 Depolymerization

**Figure 9:**
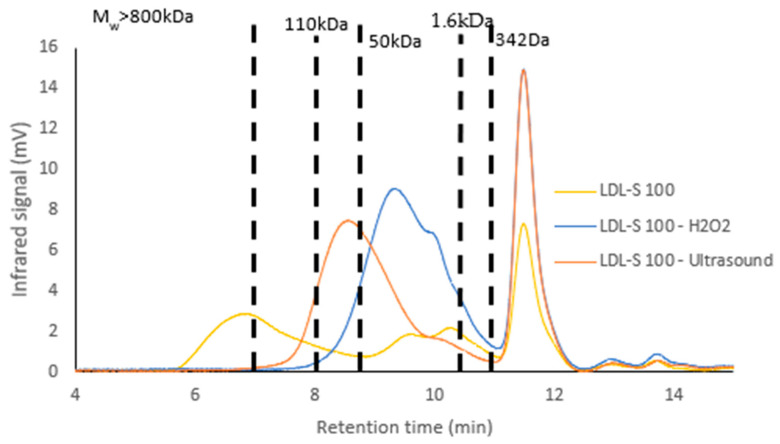
Molecular weight profile of LDL-S 100 before and after depolymerization using H_2_O_2_ and ultrasound.

LDL-S 100 was depolymerized by radical depolymerization using H_2_O_2_ (50 °C for 24 h) and ultrasound methods (50% amplitude for 24 h). The biochemical composition of the extracts was analyzed (Table 5), as well as the monosaccharide compositions and molecular weight profiles (Figure 9).

Neutral sugars remained high regardless of the depolymerization method used. On the other hand, sulfate groups were lower after depolymerization with H_2_O_2_ than with ultrasound. After either depolymerization method, the glucose content was higher (H_2_O_2_: 67.19%; ultrasound: 53.56%) than that of galactose (H_2_O_2_: 29.85%; ultrasound: 35.72%). The FTIR spectra obtained for the LDL-S 100 samples after depolymerization using both methods showed the same profile as the initial fraction, confirming the presence of ι-carrageenan in the depolymerized fractions.

LDL-S 100 contained polysaccharides with very high molecular weights (above 1.3 MDa) and low molecular weights (close to 1.6 kDa). Radical depolymerization using H_2_O_2_ produced a fraction with a molecular weight of 7.3 kDa (PD = 1.4), while ultrasound depolymerization led to a final molecular weight of 72.7 kDa (PD = 1.8).

Depolymerization of HWE1 Sc1021 and LDL-S 100 led to a significant reduction in the molecular weight of the initial fractions (from 1.3 MDa to Mw < 75 kDa) with different biochemical compositions (neutral sugar, protein, and sulfate group contents). To study the influence of the different biochemical compositions and molecular weights, HWE1 Sc1021 and LDL-S 100, along with their depolymerized fractions obtained through the hydrogen peroxide and ultrasound treatments, were used to evaluate their effects on human dermal fibroblast cells.

### 2.5. Evaluation of Polysaccharides and Low-Molecular-Weight Fractions of S. chordalis on Fibroblast Proliferation and Viability

**Figure 10:**
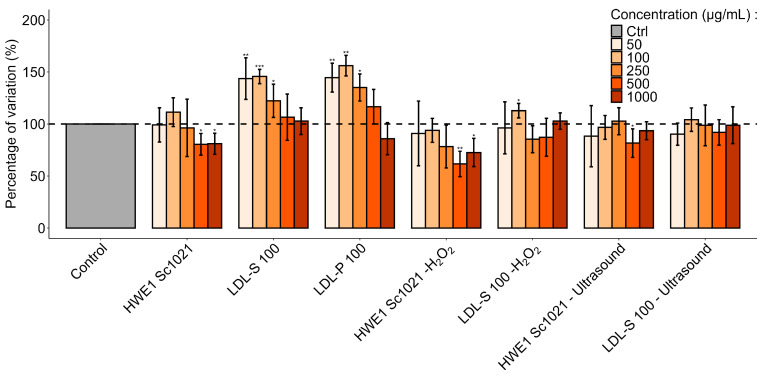
Effect of polysaccharide and low-molecular-weight fractions from *Solieria chordalis* on fibroblast proliferation, evaluated using WST-1 assays after incubating cells in presence of seaweed extracts (from 50 µg/mL to 1000 µg/mL) for 72 h (*n* = 6). Significant differences compared with negative control according to the Student’s *t*-test are indicated by * (*p* < 0.05), ** (*p* < 0.01) and *** (*p* < 0.001).

The effects of the carrageenan-rich fractions (HWE1 Sc1021, LDL-S 100, and LDL-P 100) and low-molecular-weight fractions after depolymerization using hydrogen peroxide or ultrasound on human dermal fibroblast proliferation were evaluated using the WST-1 assay (Figure 10). Fibroblast cells were treated for 72 h with 50, 100, 250, 500, and 1000 μg/mL of the different fractions.

HWE1 Sc1021 at concentrations of 500 and 1000 µg/mL produced a significant decrease in proliferation (80.52% and 81.00%, *p* < 0.05) in comparison with the untreated cells (control). The dialysis fractions LDL-S 100 and LDL-P 100 at concentrations of 50, 100, and 250 µg/mL significantly increased proliferation after 72 h of incubation to +45.69% (*p* < 0.05). The maximum proliferation was reached with 100 μg/mL of LDL-P 100 (+56.10%, *p* < 0.01). When compared to untreated cells, a significant decrease of 38.35% and 27.04% (*p* < 0.05 and *p* < 0.01) in metabolic activity was observed in the presence of carrageenan-rich fractions depolymerized using H_2_O_2_. Only LDL-S 100 after treatment with hydrogen peroxide provided a significant boost to metabolic activity (+12.83%, *p* < 0.05). The samples depolymerized with ultrasound did not influence the proliferation of human dermal fibroblasts.

**Figure 11:**
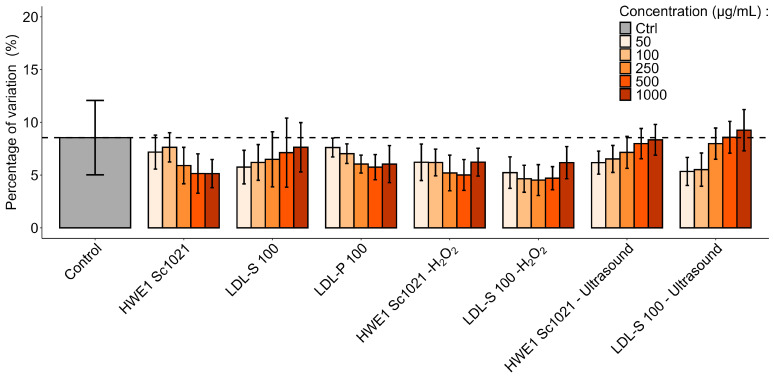
Human dermal fibroblast viability after incubation with polysaccharide and LMW fractions from *Solieria chordalis*, evaluated by lactate dehydrogenase (LDH) assay. Fibroblast cells (*n* = 5) were cultured for 72 h with five different concentrations (50 to 1000 µg/mL).

To determine whether these effects were associated with cell viability and to confirm the previous results, the potential cytotoxicity of the *S. chordalis* extracts on fibroblasts was assessed by measuring LDH (lactate dehydrogenase) release using the CytoTox 96^®^ assay after 72 h of incubation (*n* = 5) (Figure 11).

The basal LDH release in the control was calculated against the positive lysis control (100% death cell) supplied in the kit. It represents the basal level of cell death due to cell metabolism in the absence of an extract. None of the extracts showed significantly different values compared to the control (*p* < 0.05). The extracts showed no significant variation in lactate dehydrogenase release and cytotoxicity to human dermal fibroblast cells.

## 3. Discussion

*S. chordalis* sulfated polysaccharides hold considerable potential for biotechnological development. Currently, they are known to exhibit biological activities like antiviral properties [7,8,17,18]. Nevertheless, the high viscosity of these polysaccharides can limit their applications on cutaneous cells. Conversely, low-molecular-weight polysaccharides, characterized by a lower viscosity, may exhibit biological activities that could be advantageous for cosmetic applications [19].

In our study, we first produced aqueous extracts of *Solieria chordalis* samples, which were collected in October 2021 and June 2022, through maceration in order to compare their biochemical compositions. Then, HWE1 Sc1021 and LDL-S 100 were selected to produce low-molecular-weight molecules using two protocols: hydrogen peroxide and ultrasound treatments. These extracts were selected according to their biochemical compositions (molecular weight profiles and neutral sugar, sulfate group, and protein contents). In addition, the large-scale stranding of *S. chordalis* that took place on the Brittany coast in October made it possible to harvest a large biomass, leading to the extraction of a large quantity of carrageenans, which were dialyzed and depolymerized to produce LMWs.

### 3.1. Composition of the Algal Material

Different biotic and abiotic factors affect seaweed physiology, biochemical composition, as well as the properties of its bioactive compounds [20,21,22,23,24]. The contents of neutral sugars (34.36%) and proteins (14.95%) were higher in the sample from October 2021. These data are in accordance with previous studies [3,17,20], which reported neutral sugar contents of 39.70%, 27.80%, and 25.00%, respectively, in March 2012, October 2013–2015, and November 2020. In addition, these authors obtained protein contents of 22.10%, 18.50%, and 9.00%, which are consistent with the results obtained in this study. Burlot (2016) also showed that the seaweed collected in June were richer in neutral sugars (approximately 30.00%) than those collected in October (27.80%) [20]. The results of this study differ from previous findings, which can be attributed to variations in sunshine duration, water temperature, and salinity, which influence the production of neutral sugars throughout the year [1]. The high neutral sugar content in the October algae may also be explained by the conversion of summer-synthesized polysaccharides into storage sugars, such as floridean starch, to meet winter metabolic needs [25,26].

### 3.2. Extraction and Purification of Polysaccharides from Solieria chordalis

Two protocols were applied to produce aqueous extracts: traditional water extraction and water extraction at pH 9. The extraction yields ranged from 5.32% (pH 9 Sc1021) to 15.6% (HWE1 Sc0622). Previous studies showed similar hot water extraction yields of 14.6%, 3.2%, and 15.0%, respectively, for *S. chordalis* [4,8,20]. Our results are in line with these data. Choulot et al. achieved a yield of over 40.00% after 17 h of maceration in water at 50 °C [3]. The differences between the two studies may be due to the processing conditions such as extraction time, water temperature, or the use of ethanolic precipitation. The seaweed collected in June (HWE1 Sc0622) contained more hydrosoluble components (15.6%) than those collected in October (HWE1 Sc1021, 7.47%), similar to the results of the study by Burlot [20] (3.2% in October and 5.6% in June 2015). The yields of HWE1 Sc1021 and pH 9 Sc1021 were not significantly different. The pH did not seem to have a significant influence on the extraction yield, contrary to the effect on alginate yield, which requires careful monitoring of pH conditions [27]. An alkali treatment (1% KOH) was also tested in a previous study and led a polysaccharide yield of 25.8% [7]. The extraction of the HWE1 Sc1021 residue allowed for the isolation of additional water-soluble components. These results showed that a double extraction with water at 80 °C increased the extraction yield. However, maceration is an energy-intensive method, especially due to the prolonged heating required. Additionally, it involves significant water usage and is time-consuming, often leading to relatively low yields [28]. Other extraction methods such as microwave-assisted extraction (90 °C, 10 min) have shown yields close to 30% for *S. chordalis* [7]. Enzyme-assisted extraction with or without high hydrostatic pressure have achieved higher yields (from 19.8% to 34.87%, respectively) than maceration [8,29]. All the extracts contained more than 25% neutral sugars and the sulfate group content ranged from 7.73% to 16.6%, which confirmed the presence of sulfated polysaccharides. The main difference between HWE1 Sc1021 and HWE1 Sc0622 was the sulfate group content, which was higher in the seaweed collected in June. HWE2 Sc1021 was richer in neutral sugars than the other extracts, suggesting that the residue from HWE1 Sc1021 still contained polysaccharides. HWE1 Sc1021 and pH 9 Sc1021 did not show significantly different biochemical compositions. These data are different from those previously obtained with the red alga *Porphyra umbilicalis* [30]. In that study, using the pH shift technique, the protein content increased. The FTIR spectra showed the characteristic absorption patterns of ι-carrageenan with 3,6-anhydrogalactose signals (1020^−1^ and 928–930 cm^−1^) and signals from C-O-SO_3_ bonds on C2 of 3,6-anhydrogalactose (905 cm^−1^ and around 800 cm^−1^) [12,31]. They also exhibited a broad band at 1210–1250 cm^−1^, indicative of the ester sulfate. This finding agrees with the sulfate group content. It is noteworthy that the λ-carrageenan-specific signals corresponding to sulfates on carbons 2 and 6 of galactose were absent in the samples. The band at 867 cm^−1^ that is specific to the precursor ν- was not observed [7]. These data confirmed the presence of ι-carrageenan in *S. chordalis*, and are consistent with the results of the study of Deslandes et al. [32], who underlined the presence of this type of carrageenan in *S. chordalis* and in three other red algae (*Calliblepharis jubata*, *Calliblepharis ciliate*, and *Cystoclonium purpureum*). The monosaccharide compositions were similar regardless of the processing conditions. According to Usov [33], the presence of glucose can be explained by the co-extraction of floridean starch, a water-soluble *Rhodophyceae* reserve sugar [25], during polysaccharide extraction. All extracts showed a significant molecular weight peak above 1.3 MDa. This peak could correspond to an ι-carrageenan, whose molecular weight is about 944–1.6 MDa [34]. It reinforced the FTIR data and is in accordance with previous data on polysaccharides extracted from red algae with molecular weight profiles above 800 kDa [4,34,35,36,37]. A low-molecular-weight peak was also detected, which may be due to the native oligosaccharides (M_w_ ≈ 1.8 kDa) or to floridean starch (5–6 kDa). Maceration resulted in lower extraction yields compared to the other methods, but the chemical characterization of the extracts confirmed the presence of the ι-type carrageenan of interest.

Initially, HWE1 Sc1021 was dialyzed with a 100 kDa cut-off to concentrate high-molecular-weight molecules. This resulted in two phases: a supernatant (LDL-S 100) and a dialysis precipitate (LDL-P 100). Dialysis resulted in a decrease in the salt content and increase in the neutral sugar content (>30% for supernatant fraction) without any negative effects on the protein and the sulfate group contents. The precipitate from dialysis (LDL-P 100) exhibited a higher protein content compared to the supernatant (LDL-S 100). This could be attributed to the process of dialysis, wherein a portion of the protein content precipitated while the proteins bound to the polysaccharides remained in the supernatant.

### 3.3. Depolymerization of Carrageenan-Rich Fractions for LMW Production

#### 3.3.1. Study of Parameters Influencing Depolymerization Using Ultrasound

This study demonstrated the effectiveness of ultrasound in the production of low-molecular-weight fractions. Previous research has also highlighted the ability of ultrasound to significantly reduce the molecular weight of various macromolecules, such as polysaccharides from seaweeds [38,39,40,41] or from bacteria and fungi [42,43]. The importance of the choice of depolymerization parameters was underlined. Indeed, a higher amplitude (70%) achieved a greater reduction in molecular weight for the same treatment time compared with lower amplitudes. This finding was accentuated by studying the effects of ultrasound on the degradation kinetics, physicochemical properties, and prebiotic activity of polysaccharides from *Flammulina velutipes* (a fungus) [43]. Increasing the ultrasound intensity impacted the rate of polysaccharide degradation, which initially increased rapidly before slowing down. This can be attributed to the higher ultrasonic intensity enhancing the bond-breaking effect, resulting in the degradation of more polysaccharides due to high-speed vibrations and shear forces [43]. Processing time was also an important parameter to consider. The present study showed that longer treatment times resulted in a more significant molecular weight reduction. These findings are in line with the literature [40,41]. Tecson et al. [41] achieved a 96% reduction in the molecular weight of commercial κ-carrageenan (M_w_ > 1 MDa) to 4 kDa after 3 h of treatment at 85% amplitude. They also demonstrated that using ultrasound for depolymerization preserved the original structure of κ-carrageenan and its functional groups such as sulfates. Similar observations have been reported during the depolymerization of porphyran from *Porphyra yezoensis* and bacterial polysaccharides [42,44].

Ultrasound-assisted extraction is frequently employed to enhance extraction yields, with short processing times and minimal solvent usage [45,46,47]. Based on the results obtained in this study and previous research, ultrasound treatment leads to a noticeable reduction in the molecular weight of macromolecules such as algal, bacterial, and fungal polysaccharides, and other natural polymers [41,42,43,44,48]. These reductions occur during extraction using ultrasound and can induce structural changes (e.g., changes in molecular weight and biochemical composition) in the extracted compounds, thereby influencing their associated biological activities [49,50].

#### 3.3.2. Depolymerization of the Crude Extract HWE1 Sc1021 Using Hydrogen Peroxide and Ultrasound 

All crude extracts exhibited high molecular weights regardless of the extraction and dialysis conditions. To depolymerize these high-molecular-weight molecules, several methods can be applied, including chemical depolymerization (acid–alkali hydrolysis, radical depolymerization, or H_2_O_2_-induced depolymerization [51,52]), enzymatic hydrolysis (utilizing carrageenan-lyase [53]), and physical depolymerization (thermal treatment, microwave irradiation, γ-irradiation, and ultrasonication [54,55,56]).

In this study, we selected two processes: a chemical method using H_2_O_2_-induced depolymerization and a physical depolymerization using ultrasound to produce low-molecular-weight polysaccharides. Depolymerization with a radical process involves the utilization of H_2_O_2_-generated hydroxyl free radicals, which are potent oxidants, resulting in the formation of radical carbons [57,58]. Hydrogen peroxide can be regarded as a straightforward reagent due to its decomposition into oxygen and water [59]. Sonication offers precise methods for reducing the weight of polymers by breaking bonds through random scission. It involves the creation of cavitation bubbles through the passage of a sound wave with a frequency of between 20 kHz and 1 MHz through a liquid medium [60,61]. The mainly physical effects of cavitation (turbulence in the liquid flow) can lead to polymer degradation. The physical effects generally cause random splitting of the polymer chain. It is also possible to combine ultrasonic degradation with radical degradation using hydrogen peroxide. This will have the effect of causing additional degradation of molecules [48]. Both depolymerization methods were effective and reduced the molecular weight of the initial fraction HWE1 Sc1021. Hydrogen peroxide led to the production of a homogeneous fraction of LMWs (M_w_ = 7.6 kDa and PD = 1.4), similar to the study on a *Ulva* sp. [16]. Nonetheless, ultrasound treatment led to a production of 23.6 kDa molecules with a higher polydispersity index (PD = 3.3), indicating a more homogeneous mixture of molecular weights. The sulfate content (6.20%) of the hydrogen peroxide-treated sample is in accordance with the results obtained in a previous study [16]. Ultrasound depolymerization produced fractions with a higher sulfate content because it is a method that preserves the sulfate groups. These observations confirm the correlation between the depolymerization method and the structural modification of the initial components after depolymerization [62].

#### 3.3.3. Depolymerization of LDL-S 100 Using Hydrogen Peroxide and Ultrasound 

It is noteworthy that different biochemical compositions were identified in the fractions after dialysis and depolymerization. The fraction LDL-S 100 obtained after ultrasound depolymerization contained more neutral sugars (64.10%) and sulfate groups (33.68% dw) than the fraction obtained after the hydrogen peroxide treatment (34.69% and 9.72%). It would appear that the use of hydrogen peroxide led to partial desulfation of the samples, as was also shown in a previous study carried out on ulvan from a *Ulva* sp. [16]. Uronic acids were not impacted by the depolymerization method. The fractions obtained after depolymerization exhibited a reduction in molecular weight and structural groups specific to ι-carrageenan, with no modification of the constituent monosaccharides, indicating the presence of low-molecular-weight molecule-derived carrageenan, as was observed in a previous study [42]. In addition, ultrasonic depolymerization altered the solubility and viscosity of the polysaccharides [63]. However, another study showed that the use of ultrasound can modified the structure of treated molecules, thereby altering their functional properties [64]. Our results suggested that the functional groups of the native ι-carrageenan remained unchanged after depolymerization using ultrasound.

### 3.4. Effects of Carrageenan-Rich Extracts and Low-Molecular-Weight Fractions on Human Dermal Fibroblast Proliferation and Viability

The present study assessed fibroblast proliferation and viability after incubation with polysaccharide and LMW extracts from *S. chordalis* at concentrations ranging from 50 to 1000 µg/mL. The polysaccharide and low-molecular-weight extracts showed no cytotoxicity to cells after incubation with concentrations ranging from 50 µg/mL to 1000 µg/mL. These results are in accordance with those of previous studies demonstrating the non-toxicity of carrageenan on different cell types (human intestinal, liver, mammary, prostate, and cancer cell lines, Vero cells, and mouse fibroblast cells [8,65,66,67,68]). Our results showed an average increase of 45% in fibroblast proliferation for the dialyzed polysaccharide extracts. In the literature, a study on κ-carrageenans from *Hypnea musciformis* showed a decrease in cancer cell proliferation, while these same carrageenans had no effect on mouse fibroblast cells [68]. The HWE1 Sc1021 (H_2_O_2_) fraction, with oligo-molecules (M_w_ < 10 kDa), produced significant decreases in cell proliferation (20% at 500 and 1000 µg/mL). However, for the same fraction, the amount of lactate dehydrogenase released into the medium was lower than the basal level of the cells. The sample, therefore, showed no particular cytotoxic effect. The depolymerized samples (M_w_ < 25 kDa) did not increase proliferation, except for LDL-S 100 (H_2_O_2_) at 100 µg/mL. Previous studies have shown the variable influence of low-molecular-weight carrageenans (M_w_ < 30 kDa) on the proliferation of different cell types. Indeed, κ-carrageenan LMWs, obtained from commercial κ-carrageenan by enzymatic depolymerization, showed no influence on the proliferation of human umbilical vein endothelial cells [65]. Conversely, an increase in human lymphocyte cell proliferation was observed after incubation with low-molecular-weight carrageenan-rich fractions from *S. chordalis* [4].

A study on the different types of carrageenans (kappa and lambda) and hybrid carrageenans (kappa/iota, kappa/beta) highlighted the effect of structural features of carrageenan and oligo-carrageenan on their biological activities. Monosaccharide composition, molecular weight, and the number and position of sulfate groups were the main factors involved in the observed biological effects [49]. Additionally, previous studies have already shown the importance of the structure of fucoidans (presence of sulfates and reduced molecular weight) [69] or of ulvans in the metabolism of human dermal fibroblasts [70]. This could explain the various effects of the different fractions from *S. chordalis* that were observed in this study. These results highlight the potential of ι-carrageenan-rich fractions and LMW fractions from *S. chordalis* in human medicine. To our knowledge, this study shows, for the first time, the effect of the polysaccharides and LMW polysaccharides of *S. chordalis* on human dermal fibroblast proliferation and viability.

## 4. Materials and Methods

### 4.1. Algal Material

Samples of the red macroalga *Solieria chordalis* (C. Agardh) J. Agardh (Solieriaceae, Gigartinales) were collected from the beaches Port aux Moines (47°29′31.16″ N–2°49′48.14″ O) and Kercambre (47°29′21″ N–2°49′15.85″ O) at Saint-Gildas de Rhuys (Brittany, France) in October 2021 and June 2022. The algae were rinsed in fresh water, cleared of epiphytes, and then freeze-dried (Alpha 1-4 LSC, Christ). The dry matter was ground into 3 mm particles and stored at room temperature in a dark and dry place. The algal material was characterized (see Section 4.4) and used for hot water extraction.

### 4.2. Extraction and Purification of Sulfated Polysaccharides

The algal material was delipidated to remove low-polar and non-polar molecules [20,28]. Approximately 80 g of dry matter was introduced into a porous cellulose cartridge and inserted into a Soxhlet assembly. The samples were first extracted with 350 mL of 99% acetone (Fisher Chemical, Illkirch, France ) for 4 h, followed by 350 mL of a chloroform/ethanol mixture (50:50 *v*:*v*; Fisher Chemical) for 4 h, and finally with 350 mL of absolute ethanol (Fisher Chemical) for 4 h. The protocol for the hot water extraction of the delipidated dried algal matter was adapted from previous studies [16,71]. The dried algal matter (26 g) was added to 1.5 L of distilled water in an inox container heated at 80 °C and incubated for 4 h under continuous stirring (300–500 rpm). After extraction, the supernatant was precipitated with absolute ethanol (1:2 *v*:*v*, Fisher Chemical). After filtration, the precipitate was freeze-dried and stored at room temperature. The label HWE1 Sc1021 was assigned to the extracts from algae collected in October 2021, while HWE1 Sc0622 was used for those collected in June 2022 (Table 6).

The HWE2 Sc1021 label corresponds to the fraction collected after re-extraction of the residue after the first extraction. Another extraction was performed on the October algae, using the same conditions but with a pH adjustment. The pH was adjusted to 9 by adding 3 M NaOH into the extraction solution. After extraction, the aqueous part was filtered and precipitated and freeze-dried. The dry precipitate was named pH 9 Sc1021.

HWE1 Sc1021 (10 mg·mL^−1^ in distilled water) was dialyzed against distilled water for five days (cut-off of 100 kDa using Spectra/Por^®^4 Dialysis Membrane, Spectrum Laboratories, Milpitas, CA, USA); the water was replaced twice every 24 h for the three first days and once every 24 h for the next two days. The content was centrifuged at 10,000 rpm for 20 min at room temperature (Avanti J-30 I Centrifuge, Beckman, IN, USA). The supernatant and precipitate were collected, freeze-dried, and stored at room temperature. The samples were named LDL (lyophilization, dialysis, and lyophilization), followed by -S 100 for supernatant or -P 100 for precipitate.

### 4.3. Sulfated Polysaccharide Depolymerization Using Two Methods

**Figure 12:**
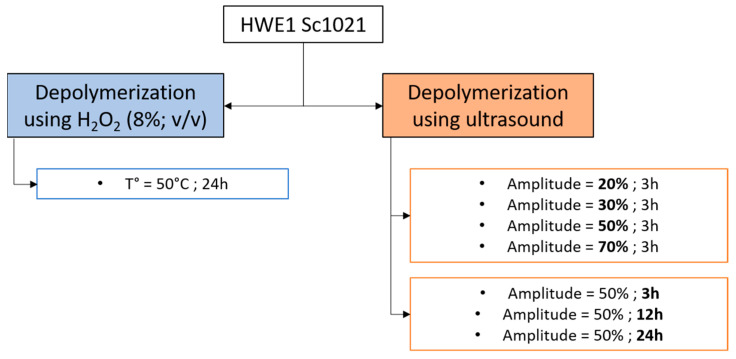
Detailed process of the depolymerization using hydrogen peroxide and ultrasound.

Depolymerization was performed on a 10 mg·mL^−1^ water solution of HWE1 Sc1021 and LDL-S 100 using two protocols: hydrogen peroxide (H_2_O_2_) and ultrasound depolymerization (Figure 12).

The depolymerization procedure using H_2_O_2_ was adapted from a previous study [16]. Briefly, polysaccharide solutions HWE1 Sc1021 and LDL-S 100 were prepared at a concentration of 10 mg·mL^−1^ in distilled water. Hydrogen peroxide (100 volumes > 30%, Fisher Scientific) was added to the solution (8% *v*:*v*) and the mixture was heated to 50 °C and incubated with stirring for 24 h. Then, the solutions were directly freeze-dried and stored at 4 °C. The ultrasonic depolymerization protocol was adapted from Tecson et al. [41]. Ultrasonic degradation was carried out using a Branson Digital Sonifier 450, Danbury, CT, USA), which was equipped with a standard horn with an output frequency of 400 W. Different amplitudes and durations were tested for the depolymerization of the HWE1 Sc1021 extract (Figure 12). All ultrasonic experiments were performed by immersing the ultrasound probe 2 cm above the bottom of a 150 mL Erlenmeyer flask immersed in an ice bath. The sample temperature was maintained at around 50 °C. LDL-S 100 was also depolymerized using ultrasound for 24 h at 50% amplitude. The fractions were freeze-dried and stored at 4 °C. In the following, the samples containing molecules with an average molecular weight of more than 10 kDa are referred to as low-molecular-weight molecules and those with an average molecular weight of less than 10 kDa are referred to as oligo-molecules (OMs).

### 4.4. Chemical and Biochemical Characterization

#### 4.4.1. Compositional Analysis of Algal Material and Extracts

The ash contents were determined gravimetrically after incineration of the samples, followed by calcining for 2 h at 585 °C. Prior to the compositional analysis, the dried samples (10 mg) were hydrolyzed for 2 h at 100 °C with 1 M hydrochloric acid (5 mL; Fisher Chemical) and neutralized with 1 M sodium hydroxide (5 mL; Fisher Chemical) for uronic acid, neutral sugar, and protein quantification. For sulfate group quantification, the dried samples (10 mg) were hydrolyzed for 2 h at 100 °C with ultrapure water (10 mL). The neutral sugar content was determined according to the phenol sulfuric method [72] using glucose as the standard. The protein content was determined using a BCA kit assay (bicinchoninic acid) and bovine serum albumin as the standard [73]. The sulfate content was measured using Azure A (3-amino-7-(dimethylamino) phenothizin-5-ium chloride), which binds to sulfate groups in polysaccharide chains, and dextran sulfate as the standard [74]. The uronic acid content was measured using the sulfamate/m-hydroxydiphenyl assay and glucuronic acid as the standard [75,76].

#### 4.4.2. High-Pressure Size-Exclusion Chromatography (HPSEC)

The samples were analyzed by using the same method as Pliego-Cortés et al. [8]. Pullulan standards from 342 Da to 1.3 MDa (PSS, Agilent, Santa Clara, CA, USA) and the samples were prepared in 0.1 M sodium nitrate (NaNO_3_), which had been previously filtered with a 0.45 µm filter, at a final concentration of 1 mg·mL^−1^ and centrifuged at 10,000 rpm for 10 min. The supernatants were collected and injected (100 µL) into an UHPLC instrument (U3000 Thermo, Scientific, Illkirch, France) equipped with a differential refractometry Iota 2 (Precision Instrument, Marseille, France) and a TSKgel^®^ GMPWXL column (7.8 mm × 30.0 cm, 13 µm) and a TSKgel PWXL guard column (6.0 × 40 mm, 12 µm, Tosoh Bioscience, Griesheim, Germany). The separation was performed using isocratic elution for 15 min with a 0.1 M NaNO_3_ solution (vacuum-filtered through a 0.45 µm filter) at a rate of 1.0 mL·min^−1^. The column temperature was kept constant at 30 °C. The chromatograms were analyzed using Chromeleon 6.8 software (Thermo Scientific, Illkirch, France).

#### 4.4.3. High-Performance Anion-Exchange Chromatography (HPAEC-PAD)

The monosaccharide composition was determined using high-performance anion-exchange chromatography (HPAEC) coupled with pulsed amperometric detection (PAD) (Thermo Dionex, Sunnyvale, CA, USA), as previously described [8,77,78]. A1 mL volume of sample (4 mg·mL^−1^) was added to 110 μL of HCl 1N and 1 mL of milliQ water in a Safe-Lock Eppendorf tube, incubated for 48 h at 100 °C, and homogenized at 700 rpm. The mix was neutralized with 110 μL of NaOH 1M and 680 μL of milliQ water. A 100 µL volume of an aqueous solution containing 2-deoxy-D-ribose (1 mg·mL^−1^) was added as an internal standard. The samples were centrifuged at 10,000 rpm for 5 min. The supernatant was used for the analysis. A 25 μL volume of the sample was injected into a CarboPac PA-1 column (4.6 × 250 mm) connected to a CarboPac pre-column (Thermo Dionex, Illkirch, France). The elution was carried out with a mobile phase composed of 82% milli-Q water and 18% 0.1 M NaOH for 30 min, followed by a gradient to 100% 0.1 M NaOH + 1 M NaOAc over 4 min. From minutes 36 to 80, the elution was performed using 82% milli-Q water and 18% 0.1 M NaOH. The column temperature was fixed at 30 °C and the flow rate was 1 mL·min^−1^. Electro-oxidation on the gold electrode corresponded to a 1000 nA current. The peaks were analyzed using Chromeleon 6.8 software (Thermo Scientific, Illkirch, France). Monosaccharides were identified and quantified by comparison to a mix of standards composed of mannitol, fucose, glucosamine, rhamnose, galactose, glucose, mannose, xylose, fructose, ribose, and glucuronic acid.

### 4.5. Fourier Transform Infrared Spectroscopy

The extract’s structural analysis employed Fourier Transform Infrared (FTIR) spectroscopy and a Nicolet iS5 spectrometer (Thermo, Madison, WI, USA), which was equipped with a universal attenuated total reflectance (ATR) sampling device containing a diamond crystal plate [8,77]. Prior to the initial analysis and at intervals of thirty minutes throughout the study, a background scan was conducted. Spectra were recorded in reflex mode within the range of 400 to 5000 cm^−1^. For each sample, sixteen scans were acquired and averaged at a resolution of 4 cm^−1^. The commercial carrageenans (SKW Biosystems, Boulogne, France) lambda (Satiagum BDC 20), iota (Siatagel DF 52), and kappa (Stiagel ME 5) were used as standards for the FTIR comparison.

### 4.6. Biological Activity

#### 4.6.1. Cell Culture

Human fibroblast cell culture and LDH cytotoxicity assays were conducted using the methods described by Fournière et al. [70]. The human dermal fibroblast samples were provided by the “Laboratoire Interactions Epithéliums Neurones” (LIEN, EA 4685) in Brest, France. The human dermal samples were obtained from skin biopsies of healthy donors undergoing abdominoplasty surgery. The study was conducted in accordance with the Declaration of Helsinki, and all patients signed an informed consent agreement. The sample collections adhered to the local ethics committee (“Comité de protection des personnes” Ouest VI) and are referenced under DC 2016-2833. Normal human dermal fibroblasts were cultured in Dulbecco’s Modified Eagle Medium F-12 (DMEM F-12, Gibco, Paisley, United-Kingdom) with 10% (*v*:*v*) fetal bovine serum (FBS, Biosera, South America), 1% antibiotic solution (*v*:*v*) (penicillin/streptomycin, 10,000 U/mL, 10 mg/mL, Gibco, Fisher Scientific, Illkirch, France), and 1% antifungal solution (*v*:*v*) (Fungizone, amphotericin B, 250 µg/mL, Gibco, Fisher Scientific, Illkirch, France), in a temperature-controlled incubator with 5% CO_2_ at 37 °C. The Ccells were sub-cultured by trypsinization with a trypsin-EDTA (ethylenediaminetetraacetic acid) (0.05%) solution (Gibco, Fisher Scientific, Illkirch, France) after reaching confluence. All experiments were performed between the 3rd and 12th passages. The cells were seeded into 96-well microplates at a density of 4000 cells/well for the WST-1 assays and LDH (lactate dehydrogenase) assays. In all the experiments, after reaching 80% confluency, the cells were incubated in DMEM with 2% FBS in the absence or presence of the samples at concentrations of 50 to 1000 µg.mL^−1^ for 72 h.

#### 4.6.2. WST-1 Assay

The WST-1 assay, which was conducted in vitro, is based on the conversion of the tetrazolium salt WST-1 (4-[3-(4-iodophenyl)-2-(4-nitrophenyl)-2H-5-tetrazolio]-1,3-benzene disulfonate) into formazan (an orange dye) by cellular mitochondrial dehydrogenases. The resulting color change is directly proportional to cell viability and proliferation in culture. After 72 h of incubation, the medium was removed, and 100 µL of the WST-1 reagent (WST-1 cell proliferation kit, Roche Diagnostics, Meylan, France; diluted 1:40 in DMEM with 2% FBS) was added to each well. Following a 45 min incubation at 37 °C with 5% CO_2_, the absorbance was measured at 450 nm and 630 nm using a microplate reader (Varioskan Lux, Thermo Fisher Scientific, Vantaa, Finland).

#### 4.6.3. LDH Cytotoxicity Assay

The LDH cytotoxicity assay is based on the measurement of lactate dehydrogenase (LDH), a stable cytosolic enzyme that is released upon cell lysis. The released LDH in culture supernatants is quantified based on the conversion of a tetrazolium salt (iodonitrotetrazolium violet) into a red formazan product. The amount of red formazan is proportional to the number of lysed cells. The LDH assay (CytoTox96^®^, Promega, Madison, WI, USA) was performed according to the supplier’s instructions. For the positive control (maximum LDH release), 10 µL of a 10× lysis solution (0.8% Triton X-100) was added for 45 min before adding the CytoTox96^®^ reagent. After incubation, 50 µL of the cell culture supernatant was transferred into a new non-sterile 96-well microplate. Then, 50 µL of the CytoTox96^®^ reagent was added. The microplate was incubated for 30 min at room temperature in the dark. The reaction was then stopped by adding 50 µL of the “Stop Solution”. LDH release was measured as the absorbance at 490 nm using a microplate reader (Varioskan Lux, Thermo Fisher Scientific, Vantaa, Finland).

### 4.7. Statistical Analysis

The results are presented as the mean ± standard deviation. Significant differences were analyzed using the Student’s *t*-test (independent, two-sided) or an ANOVA with the Tukey HSD test. However, if the data did not follow a normal distribution or if the variances were not homogeneous, the non-parametric Kruskal–Wallis test with multiple comparisons was used. All statistical analyses were performed using RStudio software version 2023.6.0.421.

## 5. Conclusions

This study investigated the extraction of carrageenan from *Solieria chordalis* using conventional hot water extraction followed by dialysis, and two depolymerization processes using hydrogen peroxide and ultrasound. Conventional hot water extraction led to different types of extracts. The pH did not significantly affect the polysaccharides obtained, but double extraction significantly improved the polysaccharide yield. Dialysis enriched the extracts with ι-carrageenan. The biochemical compositions of the extracts were similar, indicating the presence of ι-carrageenan and the presence of glucose, which was attributed to the co-extraction of floridean starch. Both depolymerization methods reduced the molecular weight of the initial polysaccharides. While the ultrasonic method was more effective in preserving sulfates, it was less efficient in reducing the molecular weight compared to the hydrogen peroxide method. The extracts exhibited no cytotoxicity on human dermal fibroblast cells after 72 h of incubation. This study suggests the potential use of these molecules in cosmetic products. Further research is required to better understand and identify the bioactive compounds (proteins, sulfates, and/or polysaccharides or LMWs) and their mechanisms of action in vitro via different biological pathways (anabolic, catabolic, and those involving the synthesis or degradation of extracellular matrix compounds).

## Figures and Tables

**Table 1:** Biochemical composition of *Solieria chordalis* harvested in October 2021 and June 2022.

Alga Sample	Ash Content	Neutral Sugars	Proteins	Sulfate Groups	Uronic Acids
	(% dw)	(% dw)	(% dw)	(% dw)	(% dw)
Sc 1021	38.15 ± 1.54 ^a^	34.36 ± 3.42 ^a^	14.95 ± 0.64	8.80 ± 0.33	4.73 ± 0.34
Sc 0622	45.00 ± 1.18 ^b^	26.68 ± 2.05 ^b^	13.20 ± 0.33	8.44 ± 0.29	4.18 ± 0.85

Sc1021: *Solieria chordalis* collected in October 2021. Sc0622: *Solieria chordalis* collected in June 2022. % dw: percentage of dry weight. Different letters in the same column represent significant differences according to *t*-tests (*p* < 0.05). Sample size: *n* = 3.

**Table 2:** Monosaccharide composition of the extracts after hot water extraction in % of total identified monosaccharides.

Sample	Gal	Glu	Xyl	GlcA
HWE1 Sc1021	22.70	60.24	2.38	6.48
HWE2 Sc1021	41.57	45.77	0.42	9.67
pH 9 Sc1021	18.24	74.04	1.24	2.95
HWE1 Sc0622	47.13	37.16	5.44	7.70

Gal: galactose; Glu: glucose; Xyl: xylose; GlcA: glucuronic acid.

**Table 3:** FTIR characteristic bands from red alga galactans [12,14].

Wavenumber (cm^−1^)	Linkage(s)/Group(s)	Unit	Type of Galactan
1730–1600	C=O aldehyde or ketone or carboxylic acid or ester group	/	/
1210–1250	S=O of sulfate esters	/	Kappa (κ), iota (ι), and lambda (λ)
1020	C-O of 3,6-anhydrogalactose	DA	Kappa (κ), iota (ι), and agar
928–933	C-O of 3,6-anhydrogalactose	DA	Kappa (κ), iota (ι), and agar
905	C-O-SO_3_ on C2 of 3,6-anhydrogalactose	DA2S	Iota (ι)
867	C-O- SO_3_on C6 of galactose	G/D6S	Lambda (λ)
848	C-O- SO_3_on C2 of galactose	G4S	Kappa (κ) and iota (ι)
825	C-O- SO_3_on C2 of galactose	G/D2S	Lambda (λ)
795–805	C-O- SO_3_ on C2 of 3,6-anhydrogalactose	DA2S	Iota (ι)

**Table 4:** Biochemical composition of HWE1 Sc1021 fraction before and after depolymerization using hydrogen peroxide and ultrasound.

	Crude Polysaccharides	Fractions after Depolymerization	
	HWE1 Sc1021	HWE1 Sc1021—H_2_O_2_	HWE1 Sc1021—Ultrasound
Neutral sugars (% dw)	32.31 ± 5.00 ^b^	29.60 ± 7.21 ^b^	43.47 ± 1.42 ^a^
Proteins (% dw)	15.64 ± 0.57 ^c^	18.64 ± 0.33 ^b^	11.62 ± 0.16 ^a^
Sulfate groups (% dw)	12.20 ± 0.84 ^c^	6.19 ± 0.49 ^b^	16.69 ± 0.14 ^a^
Uronic acids (% dw)	6.78 ± 1.14 ^c^	9.68 ± 0.67 ^b^	4.81 ± 0.45 ^a^
Molecular weight	>1.3 MDa	7.6 kDa	23.6 kDa
Polydispersity (PD)	/	1.42	3.30

% dw: percentage of dry weight. Different letters in the same column represent significant differences according to the Kruskal–Wallis test (*p* < 0.05). Molecular weights correspond to peak molecular weight.

**Table 5:** Biochemical composition of LDL-S 100 fraction before and after depolymerization using hydrogen peroxide and ultrasound.

	Dialyzed Polysaccharides	Fractions after Depolymerization	
	LDL-S 100	LDL-S 100—H_2_O_2_)	LDL-S 100—Ultrasound
Neutral sugars (% dw)	40.98 ± 7.01 ^c^	34.69 ± 9.76 ^c^	69.49% ± 2.51 ^a^
Proteins (% dw)	15.75 ± 1.06 ^c^	19.61 ± 0.71 ^a^	13.21 ± 0.42 ^b^
Sulfate groups (% dw)	11.61 ± 1.18 ^c^	9.71 ± 1.88 ^b^	33.67 ± 0.42 ^a^
Uronic acids (% dw)	5.96 ± 0.37 ^c^	13.86 ± 0.50 ^a^	8.99 ± 1.97 ^b^
Molecular weight	>1.3 MDa	7.3 kDa	72.7 kDa
Polydispersity (PD)	/	1.48	1.79

% dw: percentage of dry weight. Different letters in the same column represent significant differences according to the Kruskal–Wallis test (*p* < 0.05). Molecular weights correspond to peak molecular weight.

**Table 6:** List of extracts produced after hot water extraction.

Sample Name	Harvest Month	Extraction Method
HWE1 Sc1021	October 2021	HWE
HWE2 Sc1021	October 2021	HWE
pH 9 Sc1021	October 2021	pH adjustment followed by HWE
HWE1 Sc0622	June 2022	HWE

HWE: hot water extraction.

## Data Availability

The data presented in this study are available on request from the corresponding author without restriction.

## Supplementary Materials

The following supporting information can be downloaded at: https://www.mdpi.com/article/10.3390/md22080367/s1, Table S1: Monosaccharide composition of fractions after dialysis and depolymerization using hydrogen peroxide or ultrasound.
