# Supplementary material for: Ultrasound Depolymerization and Characterization of Poly- and Oligosaccharides from the Red Alga Solieria chordalis (C. Agardh) J. Agardh 1842
Source: Mar Drugs. 2024 Aug 13;22(8):367. doi: 10.3390/md22080367 (PMC11355074; doi:10.3390/md22080367)
Supplement: Supplementary file 1 [file marinedrugs-22-00367-s001.zip › marinedrugs-3136215-supplementary.pdf]

Table S1: Monosaccharide composition of fractions after dialysis and depolymerization using hydrogen peroxide or ultrasound

Samples name	Gal	Glu	Xyl	GlcA
LDL-S 100	32,71	46,48	7,35	7,23
LDL-P 100	21,46	61,91	4,53	8,64
HWE1 Sc1021 (1%) ; A=20; 3h	21,73	69,52	1,19	4,49
HWE1 Sc1021 (1%) ; A=30; 3h	18,17	69,85	1,44	5,73
HWE1 Sc1021 (1%) ; A=50; 3h	21,36	70,74	1,23	4,24
HWE1 Sc1021 (1%) ; A=60; 3h	19,77	69,62	1,40	5,01
HWE1 Sc1021 (1%) ; A=70; 3h	21,02	70,04	1,13	4,39
HWE1 Sc1021 (1%) ; A=50; 3h	21,36	70,74	1,23	4,24
HWE1 Sc1021 (1%) ; A=50; 12h	10,52	87,95	0,21	0,75
HWE1 Sc1021 (1%); A=50%-24h	18,71	66,59	4,21	3,99
HWE1 Sc1021_H_2O_2	18,81	67,19	4,75	6,72
LDL-S 100 H_2O_2	29,85	56,72	4,04	3,71
HWE1 Sc1021 (1%); A=50%-24h	18,71	66,59	4,21	3,99
LDL-S 100 (1%); A=50%-24h	35,72	53,56	2,49	3,42
